# Gorham-Stout disease with thoracic involvement: pathogenic mechanisms, respiratory complications, and multimodal therapies

**DOI:** 10.1186/s13023-026-04220-w

**Published:** 2026-01-30

**Authors:** Naijian Li, Xiang Le, Dawei Xu, Tanpeng Chen, Yunxiang Zeng, Jinlin Wang

**Affiliations:** 1 Department of Allergy and Clinical Immunology, State Key Laboratory of Respiratory Disease, National Clinical Research Center for Respiratory Disease, Guangzhou Institute of Respiratory Health, The First Affiliated Hospital, Guangzhou Medical University, Guangzhou, P.R. China; 2 Affiliated Qingyuan Hospital, The Sixth Clinical Medical School, Guangzhou Medical University, Qingyuan People’s Hospital, Qingyuan, P.R. China; 3 Pulmonary and Critical Care Medicine, State Key Laboratory of Respiratory Disease, National Center for Respiratory Medicine, Guangzhou Institute of Respiratory Health, The First Affiliated Hospital of Guangzhou Medical University, Guangzhou, Guangdong, P.R. China; 4 Nanshan District People’s Hospital, Shenzhen, P. R. China

**Keywords:** Gorham-stout disease, Chylothorax osteolysis lymphangiogenesis sirolimus therap

## Abstract

**Background:**

Gorham-Stout disease (GSD), also known as vanishing bone disease, is a rare osteolytic disorder characterized by progressive bone resorption and proliferation of lymphatic and vascular channels. Thoracic involvement often leads to life-threatening respiratory complications, yet clinical recognition remains delayed due to its rarity and heterogeneity.

**Objective:**

This review aims to synthesize recent advances in the understanding of GSD pathogenesis, highlight the disproportionate burden of respiratory complications-particularly chylothorax-and propose an integrated diagnostic and therapeutic framework tailored to anatomical risk profiles.

**Methods:**

A comprehensive literature review of 125 cases (2010-2025) was conducted, focusing on molecular mechanisms, clinical phenotypes, respiratory manifestations, and therapeutic outcomes. Key pathogenic pathways involving RANKL/RANK/OPG, M-CSF, VEGF, IL-6, and TNF-α were analyzed, alongside their immune-vascular interactions.

**Results:**

Respiratory involvement was observed in over 40% of cases, with chylothorax accounting for the highest mortality risk. Aberrant lymphangiogenesis, immune dysregulation, and osteoclast hyperactivation formed the mechanistic triad driving disease progression. Imaging (CT/MRI/PET) and exclusion-based diagnostics remain essential. Sirolimus-based regimens, bisphosphonates, radiotherapy, and surgical interventions-particularly thoracic duct ligation and vertebral stabilization-comprise the current multimodal strategy.

**Conclusions:**

GSD represents a clinically and mechanistically complex entity requiring early identification of respiratory threats, individualized treatment plans, and multidisciplinary coordination. Future research should prioritize biomarker discovery and prospective therapeutic trials to optimize outcomes in this rare but severe condition.

## Background

Gorham-Stout disease (GSD), also known as massive osteolysis or vanishing bone disease, is a rare, spontaneous, and progressive disorder characterized by extensive, localized bone resorption. GSD is extremely rare, with fewer than 300 cases reported worldwide to date, and no precise global prevalence has been established due to its rarity and diagnostic challenges. The underlying pathology involves the abnormal proliferation of capillaries and lymphatic vessels accompanied by fibrous tissue hyperplasia, leading to the progressive dissolution of adjacent osseous structures. While GSD can affect any skeletal site, it exhibits a predilection for the upper body, including the maxillofacial region, shoulder girdle, and critically, the thoracic cavity involving ribs, vertebrae, and sternum. This propensity for thoracic involvement carries significant clinical implications, as it predisposes patients to the development of chylothorax, a life-threatening respiratory complication resulting from disruption of the thoracic duct or lymphatic integrity due to underlying osteolysis and lymphangiomatosis. Epidemiological studies estimate that approximately 25% of GSD patients develop chylothorax, which is associated with a mortality rate as high as 46.8% [1],. According to the latest research, the mortality rate among patients with chylothorax was 12.8% [2]. Other severe complications include cerebrospinal fluid (CSF) leakage and neurological deficits [3].

The precise pathogenic mechanisms driving GSD remain incompletely elucidated. Current hypotheses strongly implicate dysregulated immune pathways and cytokine cascades. Leading research suggests that abnormal elevation of receptor activator of nuclear factor kappa-B ligand (RANKL) levels, coupled with macrophage dysfunction and heightened sensitivity to factors like macrophage colony-stimulating factor (M-CSF), may drive pathological osteoclast activation and bone remodeling [4]. Concurrently, cytokines such as vascular endothelial growth factor (VEGF) and interleukin − 6 (IL-6) are implicated in promoting the aberrant vasculolymphatic proliferation that characterizes the disease and contributes to complications like chylothorax [5, 6].

No universally accepted diagnostic criteria currently exist for GSD; diagnosis relies heavily on a combination of characteristic imaging findings, histopathological evidence of angiomatous proliferation without malignancy, and the exclusion of other causes of osteolysis, such as malignancies, infections, or metabolic bone diseases. The emergence of region-specific complications, particularly chylothorax or recurrent pleural effusions in the context of thoracic bone lesions, often serves as a critical diagnostic clue prompting further investigation for GSD [7, 8].

This review synthesizes current knowledge on GSD, with a particular focus on its respiratory implications. We delineate the hypothesized pathogenic mechanisms, evolving diagnostic approaches, the spectrum of complications (emphasizing chylothorax management), and contemporary therapeutic frameworks. The primary objective is to enhance clinical recognition and understanding of this enigmatic condition, particularly among respiratory specialists, and to optimize multidisciplinary management strategies aimed at mitigating its devastating complications, especially those impacting respiratory function.

## Methods and methods

### Literature search strategy

A systematic literature review was conducted to identify all reported cases of GSD with thoracic involvement published between January 2010 and June 2025. The electronic databases searched included PubMed, Embase, Web of Science, and the Cochrane Library. The search strategy utilized a combination of Medical Subject Headings (MeSH) terms and free-text keywords related to GSD and its thoracic manifestations: (“Gorham-Stout disease” OR “vanishing bone disease” OR “massive osteolysis” OR “Gorham’s disease”) AND (“thoracic” OR “chest” OR “chylothorax” OR “pleural effusion” OR “respiratory” OR “rib” OR “spine” OR “sternum”). The reference lists of retrieved articles and relevant review papers were also manually screened to identify additional eligible studies.

### Study selection and eligibility criteria

Studies were included if they met the following criteria: (1) reported a confirmed or highly suspected case of GSD based on established clinical, radiological, and/or histopathological criteria [8]; (2) described clear thoracic involvement, defined as osteolytic lesions in the ribs, vertebrae, sternum, or scapulae, and/or the presence of thoracic complications such as chylothorax or pleural effusion; (3) were original case reports or case series; and (4) were published in English. Reviews, editorials, non-human studies, and articles where the full text could not be retrieved or where individual patient data were not extractable were excluded.

### Data extraction and quality assessment

Data from eligible studies were independently extracted by two authors using a standardized data extraction form. Any discrepancies were resolved through discussion or by consultation with a third senior author. The extracted data included: (1) demographic information (age, sex); (2) anatomical location of bone lesions; (3) presenting symptoms and complications (e.g., chylothorax, CSF leakage, neurological deficits); (4) diagnostic modalities employed; (5) treatment regimens (pharmacological, radiotherapeutic, surgical); and (6) reported outcomes.

### Data synthesis

Due to the heterogeneity and descriptive nature of the included cases, a meta-analysis was not feasible. Therefore, data were synthesized narratively and summarized descriptively. Categorical variables were expressed as counts and percentages, and continuous variables were summarized using means with ranges or medians with interquartile ranges, as appropriate. The data synthesis aimed to characterize the clinical spectrum, complication profiles, and treatment patterns associated with thoracic GSD. The process of study identification and selection was documented following the PRISMA-ScR (Preferred Reporting Items for Systematic Reviews and Meta-Analyses extension for Scoping Reviews) framework to ensure transparency and reproducibility.

## Pathogenic mechanism

**Fig. 1:**
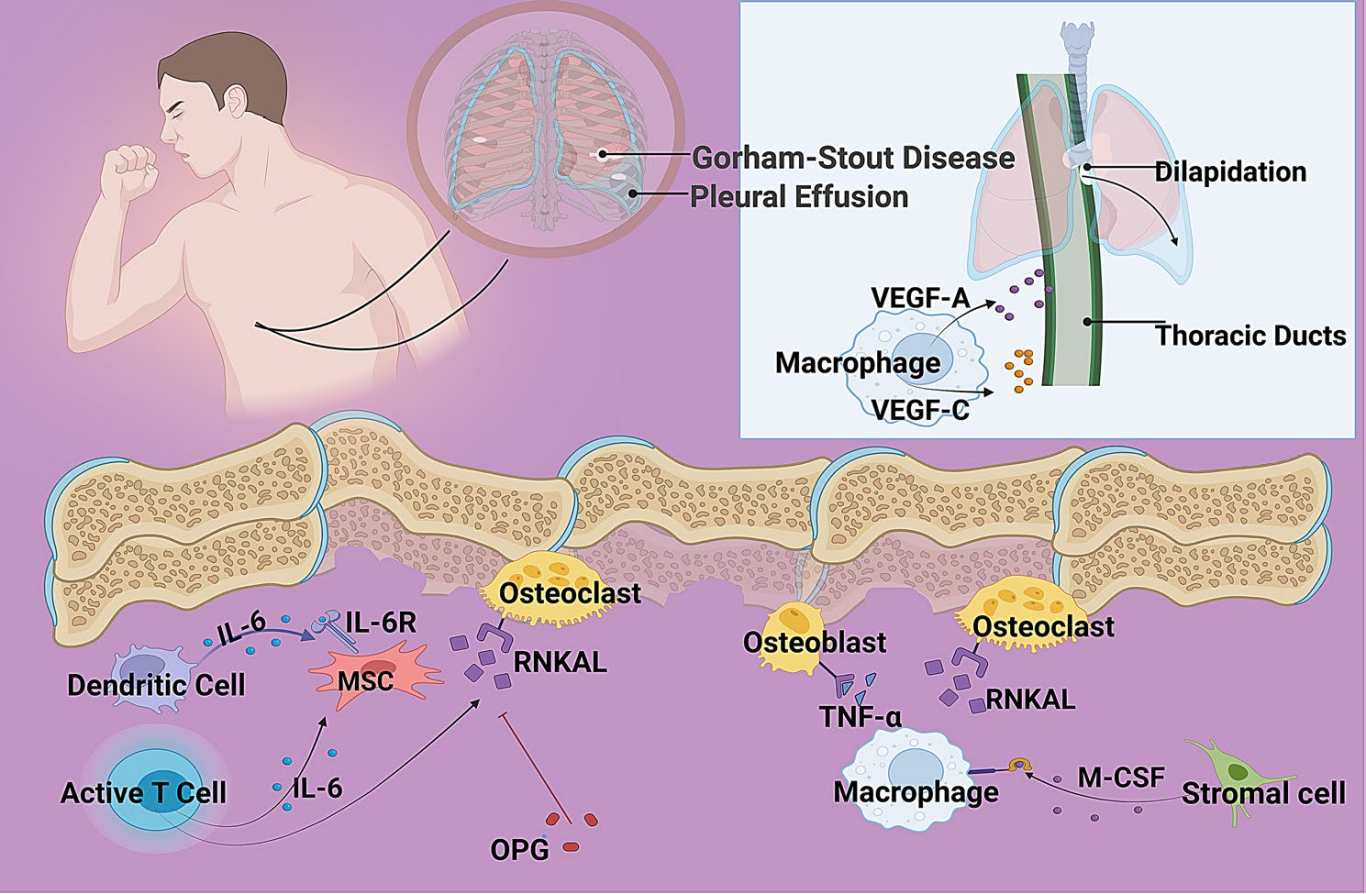
Proposed pathogenic mechanisms in Gorham-Stout disease (GSD). A schematic representation of the central molecular pathways implicated in GSD pathogenesis. Key components include: (1) RANKL-RANK-OPG axis dysregulation, resulting in excessive osteoclast-mediated bone resorption; (2) M-CSF–driven survival and priming of osteoclast precursors; (3) VEGF-C/VEGFR-3–mediated lymphangiogenesis; and (4) pro-inflammatory cytokines (e.g., TNF-α, IL-6) that amplify osteolysis and vascular proliferation via autocrine and paracrine loops

GSD is characterized by two interlinked pathological processes: progressive osteolytic destruction and aberrant proliferation of vascular and lymphatic structures. The underlying mechanisms involve dysregulation of key signaling pathways governing bone remodeling, angiogenesis, lymphangiogenesis, and immune responses (Fig. 1) [9]. Current evidence points towards immune dysfunction and cytokine dysregulation as central drivers, creating a permissive microenvironment for pathological osteoclast activation and vasculolymphatic invasion [6].

### RANKL/RANK/OPG axis

The RANKL/RANK/OPG axis is the principal signaling cascade regulating osteoclast differentiation and activity, and its dysregulation plays a critical role in GSD-associated bone loss. In the lesional stroma of GSD, pro-inflammatory cytokines-particularly IL-6 secreted by macrophages and T cells-stimulate osteoblasts, stromal cells, and activated T cells to overexpress RANKL, thereby enhancing osteoclastogenesis [6, 10, 11]. Simultaneously, the compensatory upregulation of osteoprotegerin (OPG), a decoy receptor that normally inhibits RANKL-RANK interactions, is insufficient or absent, resulting in unopposed RANKL signaling and excessive osteoclast activation [12]. This persistent activation leads to aggressive, pathological bone resorption that is uncoupled from bone formation, ultimately driving progressive osteolysis characteristic of GSD [9, 13, 14]. Clinically, this mechanism is supported by the observation that serum IL-6 levels are elevated more than sevenfold in patients with active disease, correlating with disease severity [6].

### M-CSF-Dependent mechanism

Macrophage colony-stimulating factor (M-CSF), primarily secreted by stromal cells and macrophages, is indispensable for osteoclastogenesis through its effects on myeloid progenitors. By binding to its receptor c-FMS, M-CSF promotes the survival and proliferation of osteoclast precursors while simultaneously upregulating RANK expression, thereby sensitizing these cells to RANKL-mediated differentiation [15]. In GSD, this mechanism is pathologically amplified: macrophages within lesional sites demonstrate both increased sensitivity to M-CSF and elevated local production, establishing an autocrine/paracrine loop that sustains osteoclast precursor expansion [16]. This dysregulation synergizes with RANKL signaling, markedly reducing the threshold for osteoclast formation and accelerating pathological bone resorption [15]. Moreover, macrophages play a dual role in GSD progression-not only as sources of M-CSF but also as direct contributors to the osteoclast population and as producers of pro-osteolytic and pro-angiogenic cytokines, including IL-6, TNF-α, and VEGF, which further exacerbate osteolysis and promote vascular proliferation within bone lesions [16].

### VEGF/TNF-α synergy and Lymphangiogenesis

The VEGF/TNF-α axis represents a critical intersection of inflammation, aberrant vasculolymphatic proliferation, and osteolysis in GSD. VEGF, particularly isoforms VEGF-A and VEGF-C, plays a central role in pathological vessel formation. VEGF-A, predominantly secreted by macrophages, promotes abnormal angiogenesis by enhancing vascular permeability and supporting endothelial cell survival [5]. VEGF-C, produced by both macrophages and stromal cells, is the principal driver of lymphangiogenesis; it binds to VEGFR-3 on lymphatic endothelial cells, stimulating expansion of lymphatic networks. Elevated serum VEGF-C levels in GSD patients strongly correlate with disease severity and complications such as chylothorax, indicating its pathophysiological significance [5, 6].

Tumor necrosis factor-alpha (TNF-α), secreted by activated macrophages and T cells within lesional sites, contributes to bone degradation through multiple mechanisms. It directly inhibits osteoblast differentiation and suppresses bone-forming activity while synergizing with RANKL and IL-1 to enhance osteoclast differentiation and activation-even through RANKL-independent pathways in inflamed microenvironments [6, 15]. Additionally, TNF-α acts as a potent amplifier of inflammation by upregulating IL-6, IL-1, and VEGF expression, thereby perpetuating a self-sustaining inflammatory loop [10].

Together, VEGF isoforms and TNF-α create a highly inflammatory, pro-osteolytic microenvironment in which disorganized vasculolymphatic structures invade and erode the bone matrix [5, 9]. Rather than undergoing regeneration, the resorbed bone is replaced by fibrovascular tissue, ultimately leading to the characteristic irreversible osteolysis-or “vanishing bone”-observed in GSD [9].

### Pathway crosstalk and immune dysregulation

The pathogenic mechanisms in GSD converge through extensive pathway crosstalk, embedded within a broader context of chronic immune dysregulation. A key feature is the synergistic interplay among pro-inflammatory cytokines: IL-6, markedly elevated in GSD, induces both RANKL and VEGF expression, thereby promoting osteoclastogenesis and vascular proliferation [6, 10]. TNF-α further amplifies this response by enhancing cellular sensitivity to RANKL and cooperating with IL-1 and M-CSF to drive osteoclast differentiation, even via RANKL-independent pathways under inflammatory conditions [6, 15]. Moreover, VEGF and TNF-α mutually potentiate each other’s expression while facilitating macrophage recruitment and activation, creating a self-reinforcing inflammatory milieu [10].

Macrophages emerge as central cellular hubs in this network, integrating signals from various pathways and producing a spectrum of pathogenic mediators, including RANKL, M-CSF, IL-6, TNF-α, and VEGF-A/C [16]. Their persistent activation-potentially skewed toward a pro-inflammatory M1-like phenotype-sustains both osteolysis and vascular remodeling. In parallel, the lymphatic-bone interface plays an active role in disease progression. VEGF-C-mediated lymphangiogenesis not only facilitates lymphatic expansion but also contributes directly to bone degradation: lymphatic endothelial cells have been shown to produce pro-osteolytic factors such as RANKL and to physically compromise bone structure, thus establishing a vicious cycle of bone resorption and lymphatic invasion [5].

Collectively, these findings point toward a fundamental breakdown in immune homeostasis as the root of GSD pathogenesis. Although the precise initiating events remain unclear, potential triggers include aberrant T-cell responses, chronic unresolved inflammation, and intrinsic dysfunction in macrophage activation or polarization [4, 10, 16].

## Clinical manifestations and diagnosis

Currently, there are no universally accepted diagnostic criteria for GSD; diagnosis remains one of exclusion. Given its rarity and variable clinical presentation, GSD is frequently misdiagnosed as other osteolytic conditions such as generalized lymphatic anomaly, multiple myeloma, or metastatic bone disease of unknown primary origin. As such, a multidisciplinary approach incorporating clinical, radiological, histopathological, and laboratory assessments is essential.

Heffez et al. proposed a widely referenced set of eight diagnostic criteria: (1) presence of angiomatous tissue on histopathology, (2) absence of cellular atypia, (3) minimal or absent osteoblastic response with lack of dystrophic calcification, (4) evidence of progressive and localized bone resorption, (5) non-ulcerative and non-expansile lesions, (6) no visceral involvement, (7) a radiographic pattern of osteolysis, and (8) exclusion of hereditary, infectious, neoplastic, metabolic, or immunological causes [8]. While these criteria remain useful, they are based on case reports rather than large-scale validation, highlighting the need for updated diagnostic frameworks.

Clinically, patients often present with nonspecific symptoms such as localized pain, swelling, or pathological fractures [17, 18]. Our analysis of 125 contemporary cases corroborates this, with bone pain or pathological fractures documented in 6.8% of cases (Table 1). The demographic and clinical characteristics of the 125 cases are summarized in Table 1. The cohort had a mean age of 27.6 years (range, 1 month − 82 years) and a male-to-female ratio of 1.2:1. Geographically, cases were reported from Asia (48%), Europe (32%), North America (12%), and other regions (8%), reflecting a global distribution albeit with a predominance of reports from Asian and European populations. Symptomatology depends on the anatomical site involved-for instance, mandibular lesions may cause trismus or dental pain, while spinal or thoracic involvement may lead to dyspnea, neurological deficits, or chylothorax [23]. In our case series, facial deformity was reported in 4% of cases, while trismus affected 3.2%. Critically, thoracic involvement was pervasive, with pleural effusion (encompassing chylothorax and other types) observed in 40.8% of the cohort, underscoring its central role in the disease’s morbidity. Importantly, these manifestations are often progressive and refractory to conventional therapies [41].

**Table 1:** Clinical characteristics, complications, and treatment strategies in published cases of GSD (2010–2025)

NO.	Gender	Age	Complications	Treatment process	Location of lesion
1	Female	7	Chylothorax	Unknown	Trunk and limbs [19]
2	Male	53	Facial pain、rhinorrhagia	Radiotherapy and intralesional chemotherapy injections	Left maxilla [20]
3	Male	24	Unknown	Bisphosphonates、 IFN-α-2b、 anti-VEGF therapy, mTOR inhibitors and radiotherapy and Bone graft	Left parietal skull [21]
4	Male	42	Facial depression, rash、Pleural effusion	Zoledronic acid、thalidomide、alfacalcidol	Lower jaw, ribs, fingers and phalanges [22]
5	Female	29	Pain in the right sacroiliac joint	Zoledronic acid	Right sacroiliac joint [23]
6	Female	50	Chylothorax、Cerebrospinal fluid runny nose and recurrent meningitis	Octreotide、Monogen	Mandible、Base of skull [24]
7	Female	16	Abdominal and pelvic pain	Interferon alpha 2b at 5 MIU	Abdominal and pelvic [18]
8	Male	44	Increased shoulder pain and limited mobility	Unknown	Right subscapula [25]
9	Female	4	Dorsalgia、Compression fracture	Double phosphonic acid salt, sirolimus and atenolol triple therapy	The T12 vertebrae [26]
10	Female	17	Left eye pain, inflammatory blepharoedema	Recovered on its own without medication	Anterior wall of frontal sinus [27]
11	Female	47	Maxillofacial pain、Alveolar bone resorption	Alendronate sodium、Teriparatide、deno sumab	Mandible, right frontal bone [28]
12	Female	37	Left hip pain、Spondyloarthritis（SPA）	Alendronate	Sacroiliac joint、Hip joint [29]
13	Female	3	Pleural effusion、dyspnea	sirolimus	Right humerus、scapula、clavicle [30]
14	Female	8	Pain and swelling in the left hand, and extensive swelling in the back	Bisphosphonate	Metacarpals, hamlets, triangularis, and skulls [31]
15	Female	22	Respiration distress, chylothorax、Subcutaneous hemorrhage	Sirolimus	Unknown [32]
16	Female	18	Chylothorax、Neurological impairment	Bone graft、baclofen	Left clavicle、Lumbar vertebra、pelvis [33]
17	Male	37	Pleural effusion、hypoxemia	Sirolimus	Ilium [34]
18	Female	13	Dorsalgia、tachypnea	Propranolol	Ribs、parietal bone [35]
19	Female	45	Unknown	Unknown	Left humerus [36]
20	Female	13	Chylothorax	zoledronic acid IFN-α-2b	Collarbone, scapula, sternum [37]
21	Male	14	Chylothorax	Bisphosphonates and pegylated interferon alpha-2	Clavicle [38]
22	Male	6	Cerebrospinal fluid rhinorrhea、Hearing loss	IFN-α-2b、Sirolimus	Base of skull [39]
23	Female	66	Exophthalmos in the left eye、Facial pain	Surgical excision、radiotherapy、bisphosphonate、bevacizumab	Right frontal skull [40]
24	Male	25	Recurrent cerebrospinal fluid leakage	Conservative treatment	Temporal bone [41]
25	Male	30	Cerebrospinal fluid leakage	Sirolimus	Thoracic vertebra [2]
26	Male	1	Pleural effusion, dyspnea	Sirolimus	Bilateral humerus [42]
27	Male	1	Chylothorax	IFN-α-2b	Humerus, tibia
28	Male	11 month	Chylothorax	IFN-α-2b、Bisphosphonates	Sternum、Ribs、ilium [43]
29	Male	2.5	Chylothorax	IFN-α-2b、diuretic、Bevacizumab	Pelvis, right clavicle [44]
30	Male	12	Cerebrospinal fluid rhinorrhea	Mastoid obliteration	Petrous apex [45]
31	Male	29	Odontalgia、Difficulty opening one’s mouth	RANK ligand inhibitor	Mandible [46]
32	Female	8	Pericardial tamponade、tachypnea	Sirolimus	Cervical、thoracic vertebra [47]
33	Male	5	Chiari I was deformed	Sirolimus、Bisphosphonates	Left femur humerus [48]
34	Male	19	Chylothorax	Unknown	Sternum, ribs [49]
35	Male	40	Pain in the right lateral ankle	Bisphosphonates、Bone graft	Right lateral malleolus [50]
36	Female	14	Chylothorax、Dorsal deformity	Bisphosphonates、vitamin D	Thoracic vertebra [51]
37	Male	31	Chylothorax	Zoledronic、IFN-α-2b、calcitriol、Vertebroplasty by cement augmentation	Lumbar and sacral vertebrae [52]
38	Male	3	Facial deformity, sensitive teeth	Sirolimus、Bisphosphonates、IFN-α-2b	Mandible [53]
39	Male	22	Chylothorax、Spinal deformity	pericardialwindow,chemicalthoracic duct ligation,andpleurodesis with Viscum,Thoracic spinal fusion	Thoracic vertebra [54]
40	Male	13	Chylothorax,spastic paralysis,Spinal deformity	vertebral column resection osteotomy,alendronate sodium hydrate,calcium carbonate	Thoracic vertebra [55]
41	Male	18	Pain in left arm	Bisphosphonates,Radiotherapy,Bone remodeling surgery	Humerus [56]
42	Male	32	Difficulty in neck movement,Jaw pain	Bone graft	Mandible [57]
43	Male	67	Chylothorax	zoledronic acid	Spine, ribs, pelvis [58]
44	Male	70	Walking disorder,Right hip pain	Bisphosphonates,total hip arthroplasty	Hip joint,femur
45	Male	23	Neck pain,dyspnea	Bisphosphonates,vitamin D,Mass drainage	Vertebra [59]
46	Female	15	Chylothorax,Pericardial effusion	Bisphosphonates,Thoracic catheter ligation	Sternum, ribs, ilium [55]
47	Female	27	Chylothorax	Bisphosphonates, IFN-α-2b,Vertebroplasty by cement augmentation	Femur [37]
48	Female	7	skin involvement	Bisphosphonates,Radiotherapy,IFN-α-2b	Lumbar and sacral vertebrae, pubis [57]
49	Female	33	Tooth loosening,mandible pain,Skin lesion	Radiotherapy	Mandible [58]
50	Female	22	Severe pain in the left hip	Radiotherapy,Bisphosphonates	Pelvic ring [60]
51	Male	15	Chylothorax	Bisphosphonates, IFN-α-2b,Spinal fusion surgery	Cervical vertebra [61]
52	Female	79	Facial deformity	alendronate	Mandible [62]
53	Male	58	mandibular bone loss	outleft hemimandibulectomy	Mandible [63]
54	Female	3	Cerebrospinal fluid leakage	blind sac closure of the ear,IV antibiotics	Temporal bone [64]
55	Female	30	Cervical collapse,dyspnea	Bisphosphonates	Cervical vertebra [65]
56	Male	16	cerebrospinal fluid leakage	Bisphosphonates,Filling operation	Temporal bone [66]
57	Male	26	Pleural effusion	Bisphosphonates,Radiotherapy	Rib,Left shoulder blade [42]
58	Female	11	cerebrospinal fluid leakage	IFN-α-2b,Propranolol,Repair operation	Temporal bone [39]
59	Female	22	Lower limb,paincerebrospinal fluid leakage,Chylothorax	Sirolimus, an epidural blood patch	Pelvis, femur [67]
60	Female	49	Diffuse bone pain	Unknown	Rib [68]
61	Male	51	Temporomandibular joint dislocation,Hearing loss	Unknown	Temporomandibular joint [69]
62	Female	15	Unknown	Unknown	Skull, spine [70]
63	Female	74	Persistent hip pain	Bisphosphonates,Radiotherapy	Ossa pubis [71]
64	Male	18	Intractable pleural effusion	Sirolimus,aminobisphosphonate zoledronic acid	Rib [72]
65	Male	9	Tooth loosening	Bisphosphonates,Vitamin D	Mandible [73]
66	Male	62	local tenderness in his right parietal region	Bisphosphonates,propranolol,osteogenesis	Parietal bone [74]
67	Male	27	Brainstem infarction	Bisphosphonates,Radiotherapy	Upper jaw bone,Temporal bone [75]
68	Male	37	Neck pain	Spinal fusion	Cervical vertebra [76]
69	Male	58	Neck pain, difficulty walking	Bone filling	Cervical vertebra [77]
70	Female	16	Pleural effusion	zoledronic acid,IFN-α-2b	Unknow [78]
71	Male	8	Limited mobility of the shoulder joint with pain	Unknown	Left clavicle,scapula [79]
72	Female	32	Chylothorax	bevacizu mab, zoledronic acid,enoxaparin,IFN-α-2b	Vertebrae, skull, pelvis [80]
73	Female	60	Absence of teeth,pain and hollowness in the left side of face	Refuse treatment	Mandible [81]
74	Female	43	Sternal pain, pleural effusion	Sirolimus	Rib [82]
75	Male	2	Cerebrospinal fluid leakage	mastectomy and placement of a patch	Temporal bone [83]
76	Male	45	Facial deformity	Total Joint Replacement	Mandibular condyles [84]
77	Male	53	Pain in the right foot, difficulty walking	Zoledronic acid	Metatarsal [85]
78	Male	46	Swelling of left arm	Bone filling	Left humerus [86]
79	Male	9	Chest pain,respiratory,distress dyspnea	IFN-α-2b,zoledronic acid,Propranolol	Spine,humerus,sternum [87]
80	Male	6	Chylothorax	IFN-α-2b,Bisphosphonates	Skull, vertebrae, right humerus [88]
81	Female	56	Pain and swell in elbows, ankles	zoledronic acid	Elbows, ankles [89]
82	Male	70	no	zoledronic acid	Scapula [89]
83	Male	5	Intracranial hypertension	Ketorolac,Dihydroergotamine	Mandible, cervical vertebra [90]
84	Female	60	Pathological fracture of pelvis	Bisphosphonate	Caput humeri [91]
85	Female	60	Chylothorax	Sirolimus	mandible,manubrium, cervical spine [92]
86	Female	14	Cerebrospinal fluid leakage,Bacterial meningitis	Sirolimus,Penicillin G	Base of skull [93]
87	Female	16	Chylothorax	Bisphosphonate,Sirolimus	Scapulae, bone ribs, clavicle [94]
88	Female	42	Chylothorax	Bisphosphonate,Sirolimus	Thoracic vertebra, sternum [95]
89	Female	23	Spontaneous hemothorax,Frankel B deficit	Spinal fusion	Rib [96]
90	Female	12	Back pain, difficulty walking	Pelvic fusion,Bone graft,Bisphosphonate,Sirolimus	Lumbar vertebra [97]
91	Male	47	fracture	Pamidronate,Bisphosphonate	Scapulaclavicle,humeral head [98]
92	Female	72	Limited mobility of shoulder joint	Denosumab	Glenoid scapula,humerus [98]
93	Female	66	Mild knee pain	Non-steroidal anti-inflammatory drugs, vitamin D,Bisphosphonate	Tibia, fibula [99]
94	Female	32	Chylothorax	Unknown	Sternum, thoracic vertebra [100]
95	Male	27	Difficulty walking, pain	Spinal pelvic fusion	Sacroiliac joint
96	Female	20	Chylothorax	Octreotide,Bisphosphonate	Collarbone, scapula, humerus [101]
97	Female	24	Severe lumbago,Visual impairment	Left Functional Pneumonectomy	Lumbar vertebra [102]
98	Female	23	Chylothorax,Pericardial effusion	Sirolimus,octreotide,Lenalidomide,dexamethasone,daleimumab	Clavicle, humerus, scapula [103]
99	Male	16	hip joint pain	Bone graft	Hip, femur [104]
100	Male	13	Lumbago,scoliosis	Unknown	Lumbar spine, sacrum, ilium [105]
101	Male	15	Chylothorax	IFN-α-2b,octreotide,chemical pleurodesis with OK-432	Cervical vertebra [106]
102	Male	14	Scoliosis, spinal cord compression	Bisphosphonate,Sirolimus,Instrumental correction	Rib [107]
103	Female	1	Fracture of right femur	IFN-α-2b,Sirolimus	Skull, femur, radius [108]
104	Male	76	Serous exudation of the left outer ear	a resection of the condylar process	Left condyle process [109]
105	Male	16	Diffuse chest pain	Excision, reconstruction	Sternum, ribs [110]
106	Female	18	Restriction of movement,pain	Bone graft, reconstruction	Humerus [111]
107	Male	33	Cerebrospinal fluid leakage	Dural reconstruction	Temporal bone, sphenoid bone [112]
108	Male	41	Chest pain, limited walking	immunomodulators, bisphosphonates, and supplements	Cervical vertebra, thoracic vertebra, ribs, sternum [113]
109	Female	18	Chylothorax	Unknown	Acetabulum, pubis, symphysis pubis [114]
110	Female	11	Chylothorax	Propranolol,Sirolimus	Collarbone, ribs [115]
111	Male	45	Chylothorax	Selective lymphatic embolism,Sirolimus	Ribs [116]
112	Male	48	Pain in the thigh, difficulty walking	Unknown	Femur, hip joint [117]
113	Male	21	Difficulty breathing, pleural effusion, tooth loss	Thoracocentesis,Bisphosphonate	Mandible, temporal and cheekbones [118]
114	Female	77	Progressive cranial deformity	Cranioplasty	Cranium [119]
115	Male	8	Chylothorax	Bisphosphonate,IFN-α-2b	Ribs, collarbones [120]
116	Female	9	Dyspnea, pleural effusion, bone pain	Surgical excision	Spine, ribs, tibia [121]
117	Female	11	Unilateral subjective tinnitus	Infusion therapy	Temporal bone [122]
118	Female	18	Paraplegia,Sacral swelling	Negative pressure wound	Unknown [123]
119	Female	14	Cerebrospinal fluid leakage	Dural repair, spinal fusion	Thoracic vertebra [124]
120	Female	27	Tooth loss, pain	Excision, reconstruction,bisphosphonates	mandible [125]
121	Female	37	Broken bones, abdominal pain	Radiotherapy,ibuprofen	Thoracic vertebrae, right arm [126]
122	Female	26	Spinal deformity, pain	Everolimus,Spinal fusion	Ribs, sternum [127]
123	Female	29	Facial deformity, restricted mouth opening	Bone filling	Mandible [128]
124	Female	77	Persistent resting pain	Bone graft,bisphosphonates	Femur, clavicle [129]
125	Male	1	Severe edema of left forearm,Chylothorax	Amputation above the elbow	Collarbone, ribs, femur [130]

Summary of cohort demographics and primary characteristics (*N* = 125): mean age: 27.6 years; sex ratio (M:F): 1.2:1; ethnic distribution: Asia (48%), Europe (32%), North America (12%), other (8%); most common complication: Chylothorax (26.4%)

Currently, no disease-specific biomarkers exist for GSD. However, elevated serum levels of IL-6, VEGF-A, and VEGF-C have been reported in some patients, suggesting their potential utility as adjunctive markers for disease activity [5, 6]. Routine laboratory tests, such as inflammatory markers and serum calcium levels, are generally within normal ranges and lack diagnostic specificity. Although vitamin D levels-particularly 25-hydroxyvitamin D (25-OH D)-are reported to be suboptimal in the majority of cases (ranging from 11 to 15 ng/mL, and in some cases as low as below 8 ng/mL), normal levels have also been observed in certain cases [33, 131–133]. Nonetheless, laboratory investigations remain essential for ruling out differential diagnoses such as multiple myeloma, where monoclonal gammopathy, abnormal serum free light chain ratios, and positive protein electrophoresis are typically observed [53].

**Fig. 2:**
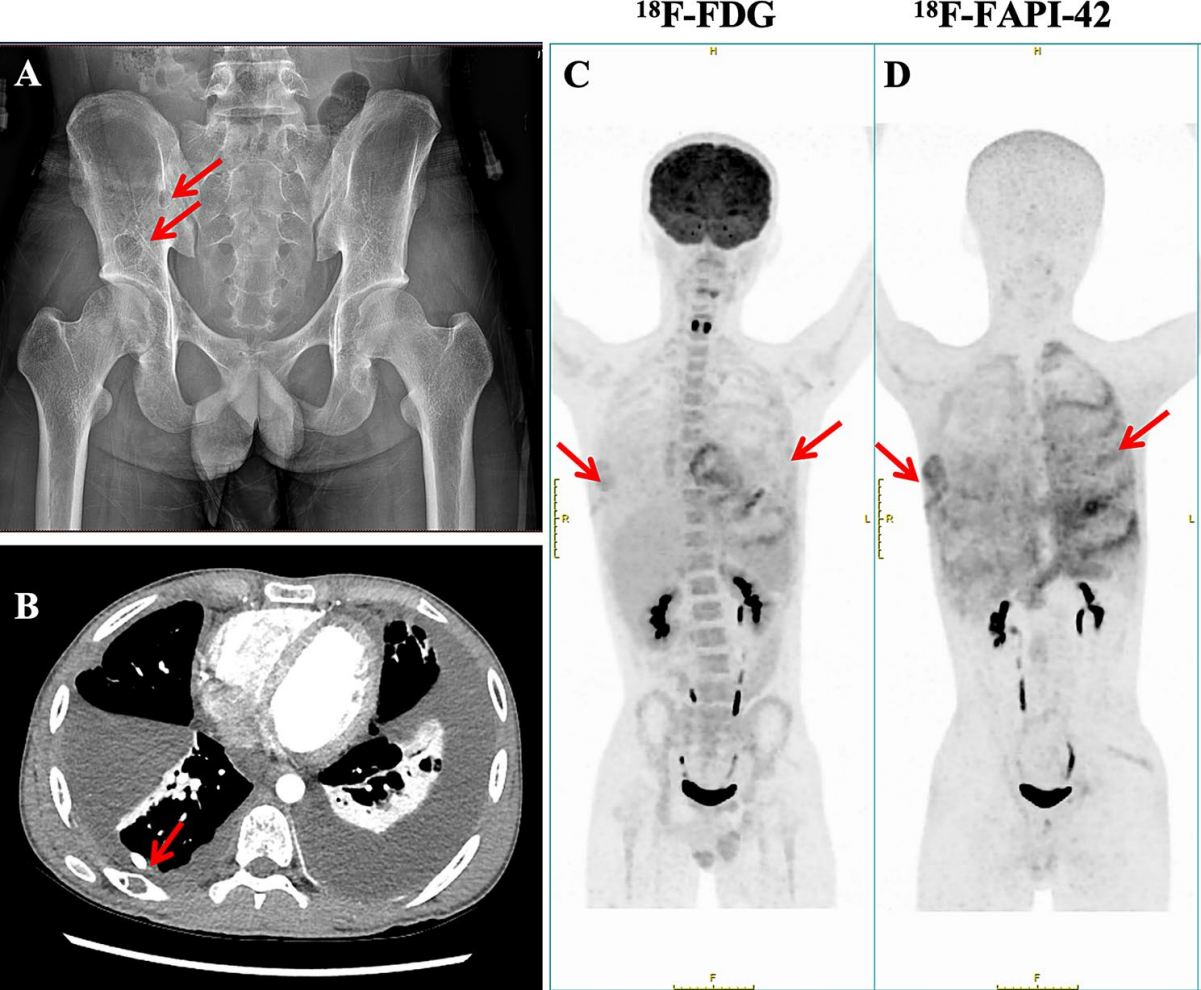
Imaging features of GSD. (A) scattered cystic fluid-attenuating lesions distributed throughout the bilateral pelvic bones. (B) multiple well-defined cystic low-density lesions involving the right posterior ribs. (C) 18F-FDG PET/CT showed mild uptake in bone loss areas, indicating regions of high glucose metabolism. (D) 18F-FAPI-42 PET/CT demonstrated higher specificity, highlighting extensive fibrotic activity in the affected bones

Imaging is pivotal in the diagnosis and monitoring of GSD. Among the 125 cases reviewed, CT and MRI were the most frequently employed modalities, effectively delineating the characteristic osteolysis and soft-tissue involvement. In early stages, conventional radiographs may show osteopenia and subtle lytic changes resembling osteoporosis. As the disease advances, more characteristic features emerge, including cortical thinning, “moth-eaten” osteolysis, and the classic “vanishing bone” appearance with bite-like erosions (Fig. 2A). Computed tomography (CT) provides superior delineation of osseous destruction, adjacent soft-tissue involvement, and hypervascular changes (Fig. 2B). Differential diagnosis from generalized lymphatic anomaly remains challenging, as the latter often presents with systemic, mass-like lymphatic lesions. Utilization of T1- and T2-weighted sequences alongside fat-suppressed imaging can aid in distinguishing these entities [91, 134–136]. Bone mineral density (BMD) testing reflects the mineral content of bone tissue, but it has limited diagnostic value for GSD. Few patients underwent BMD assessment, and the results were not entirely consistent: some exhibited reduced BMD (T-score≤−2, and in some cases even ≤-3), indicative of osteoporosis or bone loss, while others had BMD values within the normal range [133].

Nuclear imaging, though not routinely employed, offers complementary insights (Fig. 2C). Bone scintigraphy commonly reveals photopenic areas in regions of osteolysis with adjacent hyperperfusion. Emerging evidence supports the use of 18F-NaF PET/CT for identifying multifocal osteolytic lesions with partially sclerotic margins, improving anatomical delineation [137]. Conversely, 18F-FDG PET/CT typically demonstrates low or absent metabolic activity (SUVmax ~2–4), aiding in the exclusion of hypermetabolic malignancies [25].

In summary, GSD diagnosis is a process of systematic exclusion supported by radiographic hallmarks. While promising biomarkers such as IL-6 and VEGF-A/C may reflect disease activity, they lack diagnostic specificity. Integration of clinical presentation, detailed imaging-particularly CT and MRI-and targeted laboratory tests is essential to distinguish GSD from mimicking disorders. Early referral to specialized bone disease centers is recommended for comprehensive evaluation and management.

## Complications

**Fig. 3:**
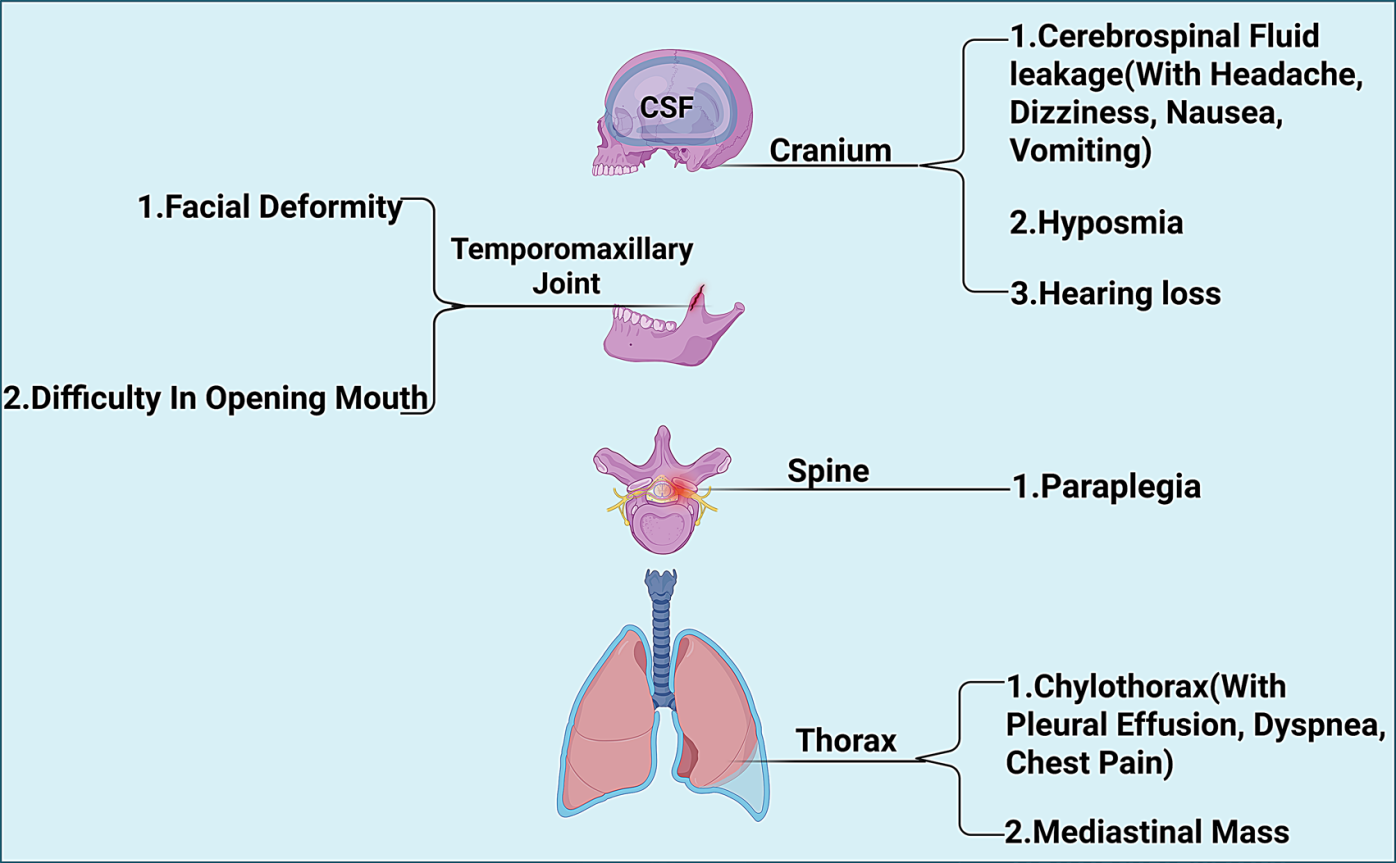
Anatomical distribution and complication patterns of GSD. clinical mapping of region-specific manifestations and associated complications based on a synthesis of over 125 published case reports (2010–2025). Common anatomical sites include the mandible, ribs, spine, and pelvis. Complications are color-coded by system, highlighting respiratory threats (e.g., chylothorax), neurological deficits (e.g., paraplegia, CSF leak), and craniofacial deformities

**Fig. 4:**
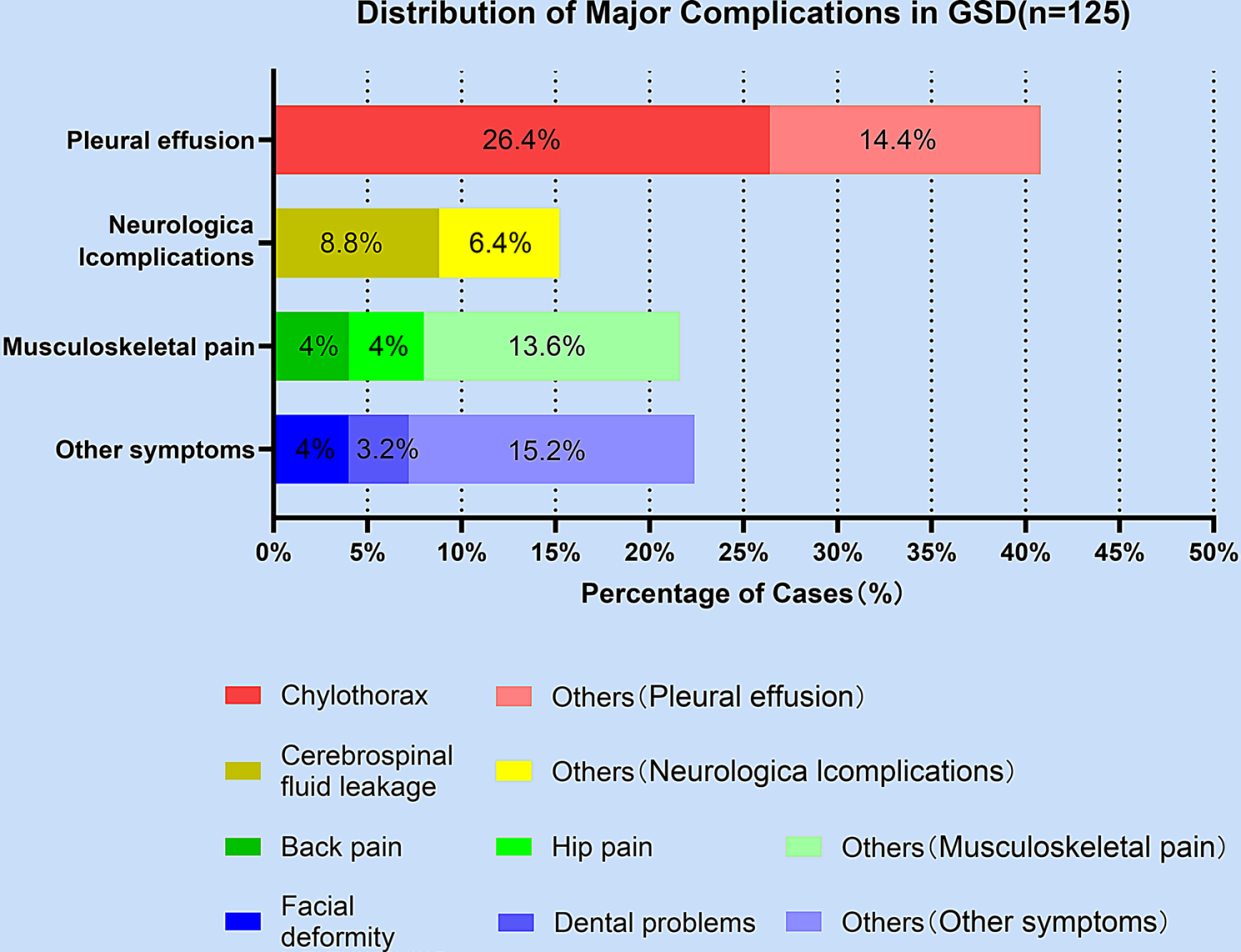
Distribution of major complications in GSD. a bar chart illustrating the proportion of various major complications among 125 GSD cases. The complications are categorized into four main groups: pleural effusion, neurological complications, musculoskeletal pain, and other symptoms. Each group is further divided into subtypes with distinct colors. Chylothorax accounts for 26.4% of the cases within pleural effusion, while other types of pleural effusion make up 14.4%. In neurological complications, cerebrospinal fluid leakage represents 8.8% and other neurological complications account for 6.4%. For musculoskeletal pain, back pain and hip pain each contribute 4%, and other musculoskeletal pain types account for 13.6%. Among other symptoms, facial deformity makes up 4% and dental problems account for 3.2%, with other unspecified symptoms comprising 15.2%. This distribution provides insights into the prevalence of different complication subtypes in GSD patients

GSD can involve virtually any skeletal region, leading to progressive osteolysis and the proliferation of abnormal vasculolymphatic structures. Among the various complications, those affecting the thoracic cavity pose the greatest threat to life and are particularly relevant to respiratory medicine (Fig. 3). Our review of 125 cases (2010–2025) provides a detailed overview of the complication spectrum, highlighting that those affecting the thoracic cavity pose the greatest threat to life (Fig. 4). The most frequent complications are detailed below.

### Chylothorax: the most lethal respiratory complication

Chylothorax represents the most serious and potentially fatal respiratory complication in GSD, occurring in approximately 25% of patients, with reported mortality rates as high as 46.8% [1]. This was the single most common major complication in our series, occurring in 26.4% (33/125) of cases, and it was consistently associated with the highest mortality risk. It results from osteolysis and lymphatic invasion into the thoracic duct or mediastinal lymphatics, causing leakage of chyle into the pleural space. Clinically, patients may present with progressive dyspnea, orthopnea, or even respiratory failure. Imaging typically reveals pleural effusion, and thoracentesis confirms chylous fluid with high triglyceride levels and lymphocytic predominance [7].

Persistent chylothorax not only impairs ventilation through lung compression but also causes severe metabolic, immunological, and nutritional derangements due to loss of lymph, proteins, and fat-soluble vitamins [25]. Mechanistically, VEGF-C–driven lymphangiogenesis and TNF-α–mediated inflammation compromise thoracic duct integrity, forming a vicious cycle of lymphatic leakage and inflammation-induced osteolysis [5, 6, 138].

### Pleural effusion and respiratory restriction

Even in the absence of chylothorax, pleural effusions may occur due to lymphovascular proliferation or direct extension of lesions into the thoracic cavity. In our cohort, non-chylous pleural effusion was observed in 14.4% of cases, indicating that a significant proportion of patients suffer from respiratory compromise even without direct thoracic duct rupture. Chronic effusion can impair pulmonary compliance, reduce lung volume, and restrict diaphragmatic motion, particularly in pediatric patients. These mechanical limitations are often under-recognized contributors to long-term respiratory disability [42, 139, 140].

### Dyspnea and chest pain

Patients with costal, sternal, vertebral, or scapular involvement may report dyspnea, pleuritic chest pain, or shallow breathing. These symptoms are multifactorial-attributable to osteolytic deformity, altered chest wall mechanics, and pleural or mediastinal invasion. Rarely, extensive rib or vertebral involvement may cause flail chest or spinal instability, further exacerbating ventilatory compromise [30, 113].

### Recurrent infections and secondary respiratory events

Complications such as CSF leakage in skull base involvement may predispose patients to recurrent meningitis and otogenic infections, which can indirectly impact respiratory status through sepsis or neurological impairment of respiratory drive [3, 39, 87]. Additionally, lymphopenia and hypoproteinemia secondary to chronic chylous loss increase susceptibility to pulmonary infections and poor healing capacity [138].

### Pulmonary parenchymal involvement

Although rare, diffuse lymphatic proliferation may extend into pulmonary interstitium in advanced cases, resembling pulmonary lymphangiomatosis. This can cause interstitial edema, impaired gas exchange, and restrictive ventilatory defects-though direct histological evidence remains limited [141, 142].

### Other systemic complications

Other debilitating complications were also quantified in our analysis. Cerebrospinal fluid (CSF) leakage, commonly associated with skull base or spinal lesions, was the second most frequent neurological complication, representing 8.8% of cases. Paraplegia secondary to vertebral osteolysis was reported in 3.4% of cases. Severe bone pain and pathological fractures in affected regions [46, 143]. This data underscores the disproportionate burden of respiratory and neurovascular involvement in GSD, emphasizing the importance of early multidisciplinary surveillance.

## Targeted pharmacotherapy in GSD

**Fig. 5:**
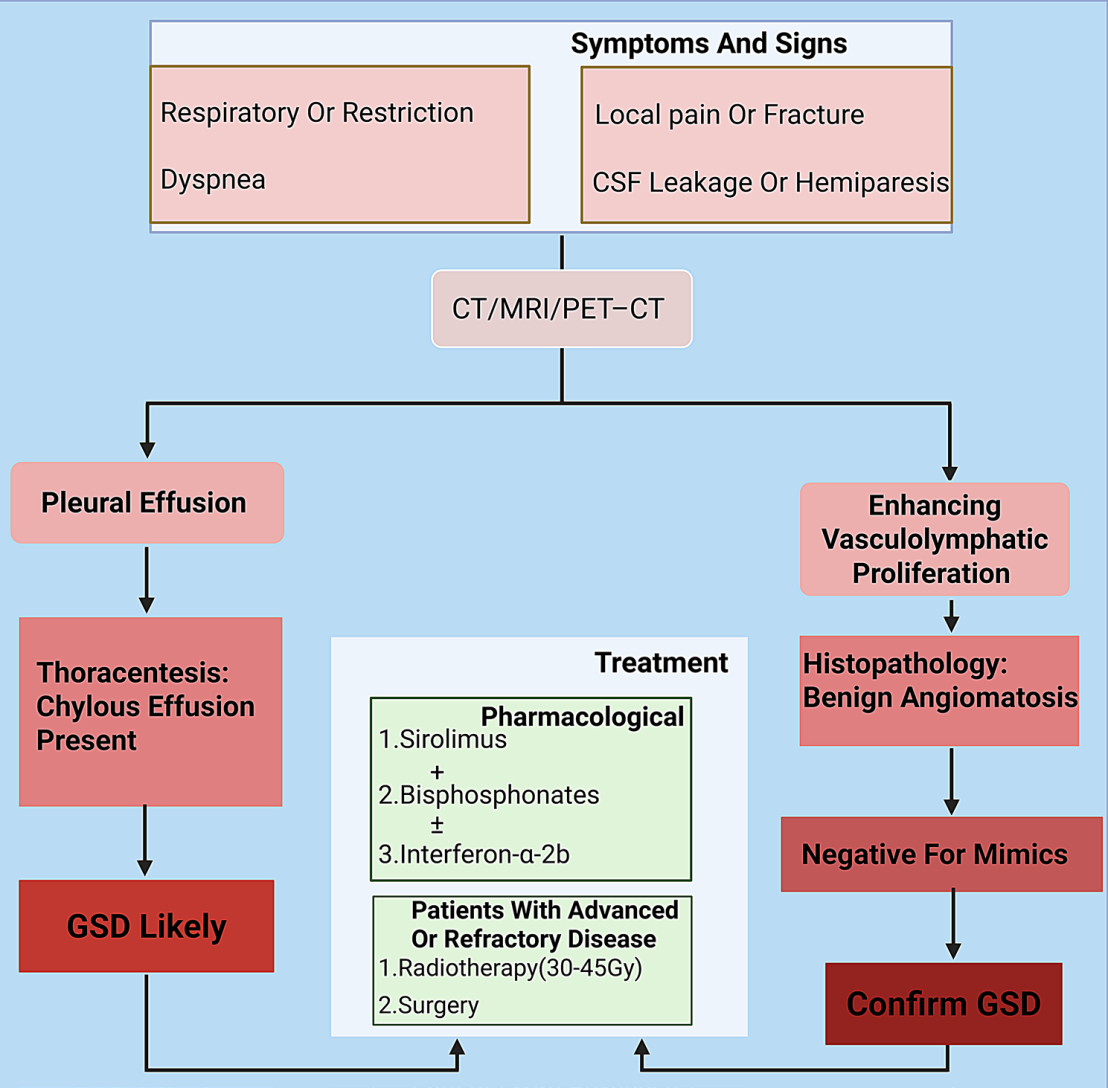
Diagnostic and therapeutic workflow for GSD. An integrated clinical algorithm outlining the diagnostic steps and treatment strategies for GSD. The pathway emphasizes early exclusion of mimickers (e.g., malignancy, infection), use of multimodal imaging (X-ray, CT, MRI, PET/CT), and tiered therapy based on disease severity. Key interventions include sirolimus-based regimens, bisphosphonates, radiation, and surgery for complications such as chylothorax and spinal instability

Pharmacological therapy remains the cornerstone of GSD management, particularly in cases where surgical intervention is not feasible or when complications such as chylothorax or diffuse lymphatic spread occur. As GSD involves both abnormal osteolysis and vasculolymphatic proliferation, pharmacological agents are typically selected to target one or both of these pathological axes. Current clinical practice rarely relies on monotherapy; most patients receive dual or triple regimens tailored to disease severity and organ involvement (Fig. 5).

### Anti-resorptive agents: bisphosphonates

Bisphosphonates are the most widely used agents in GSD and function by inhibiting osteoclast-mediated bone resorption. Zoledronic acid, alendronate, and pamidronate have been reported to reduce osteolytic activity and relieve bone pain. However, bisphosphonates exert minimal anti-angiogenic or anti-lymphangiogenic effects, limiting their use as monotherapy in progressive disease. Consequently, they are often combined with anti-angiogenic agents such as sirolimus or interferon-α-2b to address the vascular component of the disease [56, 72, 107, 144].

### mTOR inhibitors: sirolimus and Everolimus

Sirolimus, an mTOR kinase inhibitor, has emerged as a key agent for controlling pathological lymphangiogenesis. It has demonstrated efficacy in reducing lesion burden, controlling chylothorax, and improving respiratory function, especially in pediatric and early-stage patients [26, 59]. Reflecting this paradigm shift, sirolimus-based regimens were the most frequently employed systemic therapy in our reviewed cohort, used in over 50% of cases where pharmacotherapy was detailed. Clinical responses have been reported in 70–85% of treated cases, although adverse effects such as thrombocytopenia, hyperlipidemia, and mucosal ulcers may occur [145, 146]. Everolimus, a derivative of sirolimus, has also shown promise in isolated case reports, particularly when combined with surgical stabilization [127].

### Interferon-α-2b

Interferon-α-2b exhibits anti-angiogenic and immunomodulatory properties and has been used as an adjunct therapy in refractory or aggressive cases. While its efficacy appears inferior to sirolimus, it remains a reasonable second-line agent, especially in patients with vascular-dominant phenotypes or when sirolimus is contraindicated. The combination of interferon-α-2b with bisphosphonates has been reported to stabilize disease progression in several small cohorts [145].

### Other agents and adjunct therapies

Additional agents used in selected cases include:

Propranolol: A non-selective β-blocker with anti-angiogenic effects; used primarily in pediatric patients with cutaneous or mild thoracic involvement [35].

Thalidomide and lenalidomide: Immunomodulators with anti-VEGF activity, employed in experimental settings [147].

Octreotide: A somatostatin analog effective in managing chylous effusion by reducing lymphatic output [24].

Bevacizumab: A monoclonal antibody targeting VEGF-A, with sporadic use in GSD-related chylothorax and lymphangiomatosis [44].

Vitamin D and calcitriol: Supportive agents for bone metabolism, typically used alongside bisphosphonates [51].

### Therapeutic strategy and response monitoring

Optimal pharmacotherapy in GSD requires early intervention, especially in patients with rapidly progressing osteolysis or life-threatening complications like chylothorax. In clinical practice, sirolimus-based combinations (e.g., sirolimus + bisphosphonates ± interferon-α-2b) have become first-line regimens for moderate to severe disease. In our case series analysis, combination therapy was the rule rather than the exception. Sirolimus + bisphosphonates was the most common dual regimen, employed particularly in patients with concurrent aggressive osteolysis and lymphatic complications. Laboratory monitoring includes regular assessment of renal function, lipid profiles, full blood counts, and inflammatory markers. Imaging follow-up (MRI, CT, or PET/CT) is recommended every 3–6 months during active treatment phases [37, 38, 43].

## Radiation and Surgical therapy: structural stabilization and complication control

Radiation and surgical interventions play a crucial role in GSD management, particularly in cases where pharmacological therapy is insufficient or where urgent anatomical restoration is needed due to life-threatening complications such as chylothorax, CSF leakage, or structural collapse.

### Radiation therapy

Radiotherapy has demonstrated efficacy in halting disease progression by targeting the proliferative vasculolymphatic component of GSD. Its primary mechanism involves inhibition of endothelial cell proliferation and induction of vascular sclerosis. In a pooled analysis of 54 cases, Heyd et al. reported that radiotherapy with total doses of 30–45 Gy achieved disease stabilization or remission in 77–80% of patients, with no reported cases of radiation-induced malignancy [148].

Treatment planning typically involves delivering conformal radiation to the entire osteolytic region and surrounding soft tissue, with a 3–5 cm safety margin to encompass microscopic disease. Radiotherapy is especially useful in controlling localized thoracic lesions, inoperable vertebral involvement, and postoperative recurrence [60, 149]. However, long-term risks, particularly in pediatric patients, warrant cautious use and careful dosimetric planning.

### Surgical management

Surgical intervention in GSD is typically indicated for complications that threaten neurological, respiratory, or structural function, or when conservative measures fail. Surgical strategies fall into three primary categories:

Structural Stabilization and Reconstruction: In patients with pathological fractures, spinal instability, or maxillofacial deformities, procedures such as bone grafting, cement vertebroplasty, and prosthetic reconstruction are performed to restore mechanical integrity and prevent further functional loss [52, 150].

Complication Management: Surgical drainage, thoracic duct ligation, and pleurectomy are employed in refractory chylothorax to control pleural effusion and reduce respiratory compromise. Similarly, dural repair and cranial base reconstruction are indicated in cases of CSF leakage secondary to skull or spinal osteolysis [51, 88, 151].

Adjunct to Pharmacotherapy: In some cases, surgery is performed in conjunction with sirolimus or bisphosphonates to maximize disease control and reduce recurrence risk, particularly in patients with extensive disease burden.

### Clinical considerations

The success of surgical and radiotherapeutic strategies in GSD relies on several critical factors. First, precise anatomical localization using advanced imaging modalities such as CT, MRI, or PET-CT is essential for accurate preoperative planning and radiotherapy targeting. Second, adequate preoperative pharmacologic control-particularly with anti-angiogenic or anti-lymphangiogenic agents—helps reduce intraoperative bleeding risk and improves surgical outcomes. Third, postoperative monitoring, including serial imaging and biomarker assessment, is necessary to detect early signs of recurrence or residual disease. Finally, effective implementation of these interventions requires multidisciplinary coordination, typically involving orthopedic surgery, neurosurgery, thoracic surgery, radiology, and respiratory medicine [52, 56]. In patients with advanced or refractory disease, particularly those with recurrent chylothorax, progressive spinal deformity, or craniofacial instability, these modalities may be life-saving and function-preserving [51, 88].

## Treatment outcome and prognostic factors

Treatment outcomes in GSD remain highly variable, reflecting the disease’s clinical heterogeneity and lack of standardized management protocols. Among the reviewed cases, most patients received multimodal therapies combining pharmacological agents, surgical interventions, and in selected cases, radiotherapy.

Sirolimus-based regimens were the most frequently employed, especially in patients with lymphatic involvement and chylothorax, with partial or complete remission achieved in approximately 70% of reported cases. Bisphosphonates, used either alone or in combination with sirolimus or interferon-α-2b, provided symptomatic relief and radiographic stabilization in cases with dominant osteolytic lesions. Interferon-α-2b was primarily reserved for vascular-dominant phenotypes or sirolimus-refractory cases. Given the pivotal role of IL-6 in the pathogenesis of GSD, anti-IL-6 monoclonal antibodies such as tocilizumab and sarilumab represent a biologically rational therapeutic strategy. However, their application remains experimental.

Surgical interventions, including thoracic duct ligation, pleurodesis, vertebroplasty, and bone grafting, were implemented in 34% of patients, predominantly those with structural compromise or refractory chylothorax. Postoperative outcomes varied: those with isolated, surgically accessible lesions showed durable control, while diffuse or multifocal disease often relapsed.

Prognostically, thoracic involvement, particularly with persistent chylothorax, was the strongest predictor of poor outcomes, associated with increased morbidity and mortality. Additional risk factors included younger age at onset, multi-site disease, rapid skeletal collapse, and incomplete response to sirolimus. Favorable outcomes correlated with early diagnosis, monofocal disease, and prompt initiation of targeted pharmacotherapy.

## Summary

GSD is a rare osteolytic disorder characterized by progressive bone loss driven by pathological lymphangiogenesis and immune dysregulation. This review, synthesizing current knowledge and data from 125 recent cases, highlights the disease’s variable clinical features and the critical threat posed by thoracic involvement. Respiratory complications, particularly chylothorax-which affected over a quarter of our cohort-are the most life-threatening. Diagnosis remains clinical and radiological, requiring exclusion of malignancy. The analyzed cases demonstrate that current treatment strategies are inherently multimodal: sirolimus has become the cornerstone for controlling lymphatic proliferation, and is often combined with bisphosphonates to inhibit osteolysis. Surgery and radiotherapy are reserved for structural or life-threatening complications. Given the heterogeneity and severity of GSD, early recognition, individualized therapy, and multidisciplinary coordination are essential for optimal outcomes. 

## Data Availability

The raw data supporting the conclusions of this article will be made available by theauthors, without undue reservation.

## Abbreviations

CSF: Cerebrospinal fluid
CT: Computed tomography
FDG: Fluorodeoxyglucose
GSD: Gorham-Stout Disease
IL-6: Interleukin-6
M-CSF: Macrophage colony-stimulating factor
MRI: Magnetic resonance imaging
NaF: Sodium fluoride
OPG: Osteoprotegerin
PET: Positron emission tomography
RANK: Receptor activator of nuclear factor kappa-B
RANKL: Receptor activator of nuclear factor kappa-B ligand
TNF-α: Tumor necrosis factor-alpha
VEGF: Vascular endothelial growth factor
VEGF-A: Vascular endothelial growth factor A
VEGF-C: Vascular endothelial growth factor C
VEGFR-3: Vascular endothelial growth factor receptor 3
